# Validity of caregivers’ reports on prior use of antibacterials in children under five years presenting to health facilities in Gulu, northern Uganda

**DOI:** 10.1371/journal.pone.0257328

**Published:** 2021-09-16

**Authors:** Hindum Lanyero, Moses Ocan, Celestino Obua, Cecilia Stålsby Lundborg, Katureebe Agaba, Joan N. Kalyango, Jaran Eriksen, Sarah Nanzigu

**Affiliations:** 1 Department of Pharmacology and Therapeutics, Makerere University College of Health Sciences, Kampala, Uganda; 2 Mbarara University of Science and Technology, Mbarara, Uganda; 3 Department of Global Public Health, Karolinska Institutet, Stockholm, Sweden; 4 Infectious Diseases Research Collaboration, Kampala, Uganda; 5 Department of Pharmacy, Makerere University College of Health Sciences, Kampala, Uganda; 6 Clinical Epidemiology Unit, Makerere University College of Health Sciences, Kampala, Uganda; 7 Department of Infectious Diseases, South General Hospital, Stockholm, Sweden; University of Washington, UNITED STATES

## Abstract

**Introduction:**

Given the frequent initiation of antibacterial treatment at home by caregivers of children under five years in low-income countries, there is a need to find out whether caregivers’ reports of prior antibacterial intake by their children before being brought to the healthcare facility are accurate. The aim of this study was to describe and validate caregivers’ reported use of antibacterials by their children prior to seeking care at the healthcare facility.

**Methods:**

A cross sectional study was conducted among children under five years seeking care at healthcare facilities in Gulu district, northern Uganda. Using a researcher administered questionnaire, data were obtained from caregivers regarding reported prior antibacterial intake in their children. These reports were validated by comparing them to common antibacterial agents detected in blood and urine samples from the children using liquid chromatography with tandem mass spectrometry (LC-MS/MS) methods.

**Results:**

A total of 355 study participants had a complete set of data on prior antibacterial use collected using both self-report and LC-MS/MS. Of the caregivers, 14.4% (51/355, CI: 10.9–18.5%) reported giving children antibacterials prior to visiting the healthcare facility. However, LC-MS/MS detected antibacterials in blood and urine samples in 63.7% (226/355, CI: 58.4–68.7%) of the children. The most common antibacterials detected from the laboratory analysis were cotrimoxazole (29%, 103/355), ciprofloxacin (13%, 46/355), and metronidazole (9.9%, 35/355). The sensitivity, specificity, positive predictive value (PPV), negative predictive value and agreement of self-reported antibacterial intake prior to healthcare facility visit were 17.3% (12.6–22.8), 90.7% (84.3–95.1), 76.5% (62.5–87.2), 38.5% (33.0–44.2) and 43.9% (k 0.06) respectively.

**Conclusion:**

There is low validity of caregivers’ reports on prior intake of antibacterials by these children. There is need for further research to understand the factors associated with under reporting of prior antibacterial use.

## Introduction

Antibacterial agents are used to treat a wide range of bacterial infections and are essential lifesaving medicines. They are the most commonly used medicines in Sub-Saharan Africa due to the high prevalence of infectious diseases [1]. Used correctly, they deliver enormous benefits to the health of the population worldwide [2].

Antibacterials are, according to the national drug policy of Uganda, prescription only medicines [3]. However, they are readily accessible and affordable to most patients within the communities in Uganda, not only as prescription medicines as they can often also be obtained over-the-counter especially in private medicine outlets [4]. The relative ease with which communities access these medicines poses several challenges for antibacterial stewardship [4]. The majority of caregivers in low-income countries initiate treatment of their children at home [5]. The use of antibacterials prior to hospital visits is common, especially in low-income countries, and may influence patient treatment outcomes. According to a study in Nigeria, 85% of patients reported to have self-medicated before coming to the health facility and antibacterials were among the most common medicines used [6]. A study in Uganda reported that 62.2% of patients had used antibacterial agents prior to coming to health facility [4]. Another study done in Haiti to assess self-medication among patients presenting at an out-patient department found that 45.5% practiced self-medication with antibacterials [7].

Caregivers’ ability to report antibacterial intake prior to coming to a health facility is crucial for appropriate prescription of medicines at the health facility. Self-reports have been shown to have low validity as they are prone to recall bias and social desirability bias. Respondents normally provide information that conforms to their perceived expectations of the health workers or researchers [5, 8]. A study carried out in Uganda in 2009 reported a limited validity of caregivers’ reports of use of sulfamethoxazole, chloroquine and sulfadoxine in their children prior to arrival to the hospital [5]. Similarly, a study from Tanzania reported that 97% of the children without history of prior chloroquine treatment had detectable levels of chloroquine in blood [9]. Another study in Ghana reported a high prevalence (64%) of antibacterials detected in urine samples of patients compared to the self-reported use (13%) [10].

To our knowledge no study has validated caregivers’ reports of intake of antibacterials in children under five years in rural communities in low resource settings. In this study we describe and validate caregivers’ reported use of antibacterials by their children under five years for treatment prior to seeking care at the healthcare facility.

## Materials and methods

### Ethics statement

The protocol was reviewed and approved by the Makerere University School of Biomedical Sciences Research and Ethics Committee (reference SBS-570) and the Uganda National Council of Science and Technology (reference HS235ES) (S1 Appendix). Administrative clearance was obtained from the healthcare facilities where the study was conducted. Written informed consent was obtained from caregivers of children under five years prior to data collection (S2 Appendix).

### Study design and setting

A cross-sectional study was conducted among children under five years and their caregivers in healthcare facilities in Gulu district, northern Uganda. Gulu is located about 360 km from the capital city Kampala. In Uganda, the lowest level of the district-based healthcare system consists of the village health teams/community medicine distributors, which constitute level 1 of health care. This is operated by members of the community who can read and write at least in the local language of the community. The next level is health centre II which is operated by a professionally trained nurse with a diploma and is intended to serve 5000 patients. This is followed by health centre level III which is operated by a professionally trained clinical officer with a diploma in clinical medicine and intended to serve 10,000 patients. Above health centre level III is health centre level IV and then district hospitals headed by medical officers with a basic degree in medicine and surgery and intended to serve about 100,000 patients. Regionally there are regional referral hospitals where patients are referred to from the district hospitals. The regional referral hospitals are expected to have specialist health professionals covering the major disciplines such as surgery, internal medicine, and paediatrics. At the top of the health care system are the national referral hospitals [11]. Gulu district has a total of 19 health centre level II, 10 health centre level III, one health centre level IV, 31 registered pharmacies and 135 licensed drug shops [12–14]. This study was carried out in three health centre level III and one health centre level IV. These healthcare centers were purposively selected because they serve the greatest number of patients in the out-patient departments in Gulu district. The most common diseases in children under five years seeking care at healthcare facilities in this area include; malaria, diarrhea, pneumonia, acute childhood malnutrition and HIV/AIDS [15–17].

### Study population

Sick children under five years and their caregivers seeking care at the four healthcare facilities were included in the study after caregivers’ consent. Children who were brought to the health center by caregivers who did not take care of the children from the onset of the current illness were excluded from the study. Children who had come for review or continuation of treatment for current illness were also excluded from the study.

### Sample size

The sample size was computed based on formula for estimation of sample size for a single proportion [18]. Assuming that the proportion of children getting antibacterial treatment prior to health facility visit was 50%, in order to have a 95% confidence interval and a 5% margin of error, the minimum sample size needed was set to 385. The number of children sampled from each facility was determined from the volume of patients at the health facility using proportionate sampling.

### Sampling procedures

The patients were selected by systematic random sampling. On each of the data collection dates the first patient to be recruited into the study was randomly selected by having a blindfolded data collector walk around in the waiting area and point at a random patient among the patients waiting in line to be seen by the healthcare worker in the outpatient department. Thereafter, every fourth patient in line towards the entrance of the healthcare workers room was selected for recruitment. In the event that the selected patient was above five years of age, they were skipped and the next patient recruited while maintaining the sampling interval. Approximately 10 days were spent collecting data in each healthcare facility.

### Data collection

An interviewer administered questionnaire was used for data collection. The questionnaire was pre-tested on caregivers of 30 children in outpatient departments of Gulu regional referral hospital. This tool was adapted from a tool used to collect data on prevalence and predictors of prior antibacterial use among patients presenting to hospitals in northern Uganda in a previous study [4], it was written in English and translated to Acholi (the most common local language spoken in the study area).

The data collection team was divided into four groups each comprising of two people, one pharmacy technician (health professional with diploma in pharmacy) and a laboratory technician. The pharmacy technician conducted interviews while the laboratory technician collected the blood and urine samples.

Information on the following variables was collected; sub-county of residence, age of child, age of care-giver, sex of child, sex of caregiver, whether medication was given to child before coming to the healthcare facility since the onset of this current illness, the type and source of the medicine, and the person who recommended the medicine. In case the caregiver did not know the name of the medicine, the interviewer asked them to describe it or show the packing material if at all they had come with it to the health center. Each interview lasted about 20 minutes per patient.

#### Sample collection and transportation

Two hundred microlitres (200μL) of blood was collected from the fingertips of children under five years using a 200μL micro-pipette with ethylenediamine tetra-acetic acid (EDTA), and spotted on a filter paper and left to dry for 3 hours in room temperature. After the blood had dried on the filter paper, each filter paper was put in a separate plastic zip bag with a desiccant and transported to the laboratory for analysis.

Urine samples were collected in sterile wide mouth containers. In the very young children who couldn’t void in the wide mouth containers, urine samples were collected by placing a thick layer of cotton wool inside the child’s nappy and squeezing the urine in the urine sample bottles. Two hundred microlitres (200μL) of urine was collected from the wide mouth containers using a plastic pipette and spotted on a filter paper and left to dry for 3 hours at room temperature. After the urine had dried on the filter paper, each filter paper was put in a separate plastic zip bag with a desiccant and transported to the laboratory for analysis. The dried blood spot (DBS) and dried urine spot (DUS) samples obtained from patients were stored at -20°C and -80°C respectively until analysis.

#### Extraction and analysis of antibacterials in dry blood spot and dry urine spot samples

The whole diameter disk (containing 200μl of blood or urine) was cut out from each DBS and DUS. The cut disc was placed in an Eppendorf tube (1.5 mL capacity) and mixed with 1000 μL of methanol (20%) and acetonitrile (80%). The sample was vortex-mixed twice for 20 s at 10-min intervals and then centrifuged at 3500 revolutions per minute (RPM) for 5 minutes. After the extraction period, the filter paper was removed, and 500 μL of the extract was transferred into an auto-sampler vial to be injected onto the LC-MS/MS system for analysis.

A simple, fast, sensitive and selective qualitative LC-MS/MS method for identification of fifteen (15) antibacterials in DBS and DUS was used for analysis (S3 Appendix). The limit of detection for the different antibacterials were: amoxicillin (1.34 ng/mL), ampicillin (0.001 ng/mL), penicillin G (0.005 ng/mL), penicillin V (0.03 ng/mL), cloxacillin (0.2 ng/mL), cephalexin (0.22 ng/mL), sulfamethoxazole (0.95 ng/mL), trimethoprim (0.52 ng/mL), erythromycin (1.1 ng/mL), ciprofloxacin (0.1 ng/mL), tetracycline (0.14 ng/mL), clarithromycin (1.4 ng/mL), metronidazole (0.0004 ng/mL), chloramphenicol (0.0001ng/mL) and azithromycin (0.22 ng/mL).

Data on key pharmacokinetics properties that may have affected the interpretation of our results, have been presented in the supporting information section (S1 Table), and these include: clearance, terminal half-life, percentage of medicine excreted in urine, time to peak plasma concentrations and volume of distribution.

### Data management

Double data entry was done using Epi-Data 3.1 software for both the questionnaire and laboratory data. The two datasets were reconciled by comparing them for each field in the questionnaire and laboratory result, in case of any discrepancies, the corresponding questionnaire or patient laboratory record was checked to establish the correct entry. Data were then imported into Stata 14/IC (Stata Inc., Texas USA) for analysis.

### Statistical analysis

Descriptive statistics were presented using median and interquartile range (IQR) for continuous variables or frequencies and proportions for categorical variables. The dependent variables, treatment of child with antibacterials prior to healthcare facility visit as reported by their caregiver and detectable antibacterials in DBS or DUS samples, were summarized as proportions. In order to adjust for potential biases associated with point estimates from the sampling design, we used svy commands in stata to compute proportions and respective 95% confidence intervals. Pearson’s chi-square test was used to assess associations for the categorical variables. In order to validate caregivers’ reported use of antibacterials, sensitivity, specificity, positive predictive value (PPV), negative predictive value (NPV), prevalence, agreement and kappa coefficient were calculated. Laboratory results for detection of antibacterials in dry blood spot or dry urine spot samples were considered as the gold standard and caregivers’ reports of use of antibacterials prior to health facility visit were considered as the test results.

## Results

### Socio-demographic characteristics of the caregivers and children under five years

Of the 385 sampled children, 355 (92.2%) had data on both caregiver’s report on antibacterial use prior to health facility visit and results from urine and blood analysis and were thus included in the analysis. The 30 (7.8%) observations were dropped because they were missing blood analysis data. Over half (53.2%, n = 189) of the children were female. The median age of the children was 29 (IQR: 16–46) months. The majority (96.1%, n = 341) of the caregivers were female. The median age of the caregivers was 25 (IQR: 21–31) years. About half (53.2%, n = 189) of the children attended a healthcare facility located in a rural area. (Table 1).

**Table 1:** Socio-demographic characteristics and prevalence of antibacterial use in children under five years prior to health facility visit as reported by caregivers of children under five years in rural communities of Gulu district, northern Uganda (August, 2019).

Characteristics	Description	Respondent’s Frequency (%)	Proportion of reported antibacterial use, n (%)	95% CI	P-value (Pearson’s chi-square test)
**Overall**		355 (100)	51 (14.4)	10.9–18.5	
**Sex of child**	Male	166 (46.8)	22 (13.3)	8.9–19.4	0.575
	Female	189 (53.2)	29 (15.3)	10.9–21.3	
**Location of health facility**	Urban	166 (46.8)	36 (21.7)	16.0–28.6	<0.001
	Rural	189 (53.2)	15 (7.9)	4.8–12.8	
**Age of child (months)**	1–12	64 (18.0)	6 (9.4)	4.2–19.5	0.119
** **	13–36	176 (49.6)	32 (18.2)	13.1–24.6	
** **	37–59	115 (32.4)	13 (11.3)	6.7–18.6	
**Age of child caregiver (years)**	13–22	121 (34.1)	15 (12.4)	7.6–19.6	0.936
** **	23–32	168 (47.3)	27 (16.1)	11.2–22.5	
** **	33–42	51 (14.4)	7 (13.7)	6.6–26.3	
	43–52	7 (2.0)	1 (14.3)	1.7–62.3	
** **	≥ 53	8 (2.2)	1 (12.5)	1.5–57.5	
**Sex of child caregiver**	Male	14 (3.9)	1 (7.1)	0.9–39.0	0.432
** **	Female	341 (96.1)	50 (14.7)	11.3–18.9	
**Source of antibacterials**	Home cabinet		11 (23.9)	12.5–38.8	<0.001
	Public health facility		9 (37.5)	18.8–59.4	
	Private clinics		8 (30.8)	14.3–51.8	
	Drug shops		18 (35.3)	22.4–49.9	
	Retail shops		4 (30.8)	9.1–61.4	
	Traditional healers		1 (50)	1.3–98.7	
**Antibacterials recommended by**	Caregiver		11 (31.4)	16.9–49.3	0.253
	Other household member		3 (37.5)	8.5–75.5	
	Friend/neighbor		1 (25.0)	0.6–80.6	
	Doctor/nurse		17 (34.0)	21.2–48.8	
	Drug seller/pharmacist		18 (29.0)	18.2–41.9	
	Traditional healer		1 (33.3)	0.8–90.6	
**Age of child (months), median (IQR) 29 (16.46)**					
**Age of child caregiver (years), median (IQR) 25 (21.31)**					

n: Sample size; CI: Confidence Interval; %: Percentage; IQR: Interquartile range

### Prevalence of antibacterial use prior to coming to the health facility as reported by caregivers of children under five years

Out of the 355 children under five years who were included in the analysis, 51 (14.4%, CI: 10.9–18.5) were reported by the caregivers to have been treated with antibacterials prior to coming to the healthcare facility. Of these 51 children, the prevalence of antibacterial use was higher in those from urban areas (21.7%, CI: 16.0–28.6) and in those who got antibacterials from public health facilities (37.5%, CI: 18.8–59.4) (Table 1).

### Prevalence of antibacterials detected in blood and urine samples of children under five years

Of the 355 children under five years who were included in the analysis, 226 (63.7%, CI: 58.4–68.7) had detectable levels of antibacterials in urine or blood in the samples taken upon arrival to the healthcare facility (Table 2).

**Table 2:** Prevalence of antibacterials detected in blood or urine samples of children under five years in rural communities of Gulu district, northern Uganda (August, 2019).

Characteristics	Description	Respondent’s Frequency (%)	Proportion of antibacterial detected, n (%)	95% CI	P-value (Pearson’s chi-square test)
Overall		355 (100)	226 (63.7)	58.4–68.7	
Sex of child	Male	166 (46.8)	108 (65.1)	57.5–71.9	0.608
	Female	189 (53.2)	118 (62.4)	55.3–69.1	
Location of health facility	Urban	166 (46.8)	103 (62.0)	54.4–69.1	0.554
	Rural	189 (53.2)	123 (65.1)	57.9–71.6	
Age of child (months)	1–12	64 (18.0)	45 (70.3)	57.9–80.3	0.389
	13–36	176 (49.6)	112 (63.6)	56.2–70.4	
	37–59	115 (32.4)	69 (60.0)	50.7–68.6	
Age of child caregiver (years)	13–22	121 (34.1)	83 (68.6)	59.7–76.3	0.164
	23–32	168 (47.3)	99 (58.9)	51.3–66.2	
	33–42	51 (14.4)	36 (70.6)	56.6–81.5	
	43–52	7 (2.0)	5 (71.4)	29.7–93.7	
	≥ 53	8 (2.2)	3 (37.5)	11.4–73.6	
Sex of child caregiver	Male	14 (3.9)	8 (57.1)	30.7–80.1	0.605
	Female	341 (96.1)	218 (63.9)	58.7–68.9	

n: Sample size; CI: Confidence Interval; %: Percentage

#### Most commonly used antibacterials

**Fig 1:**
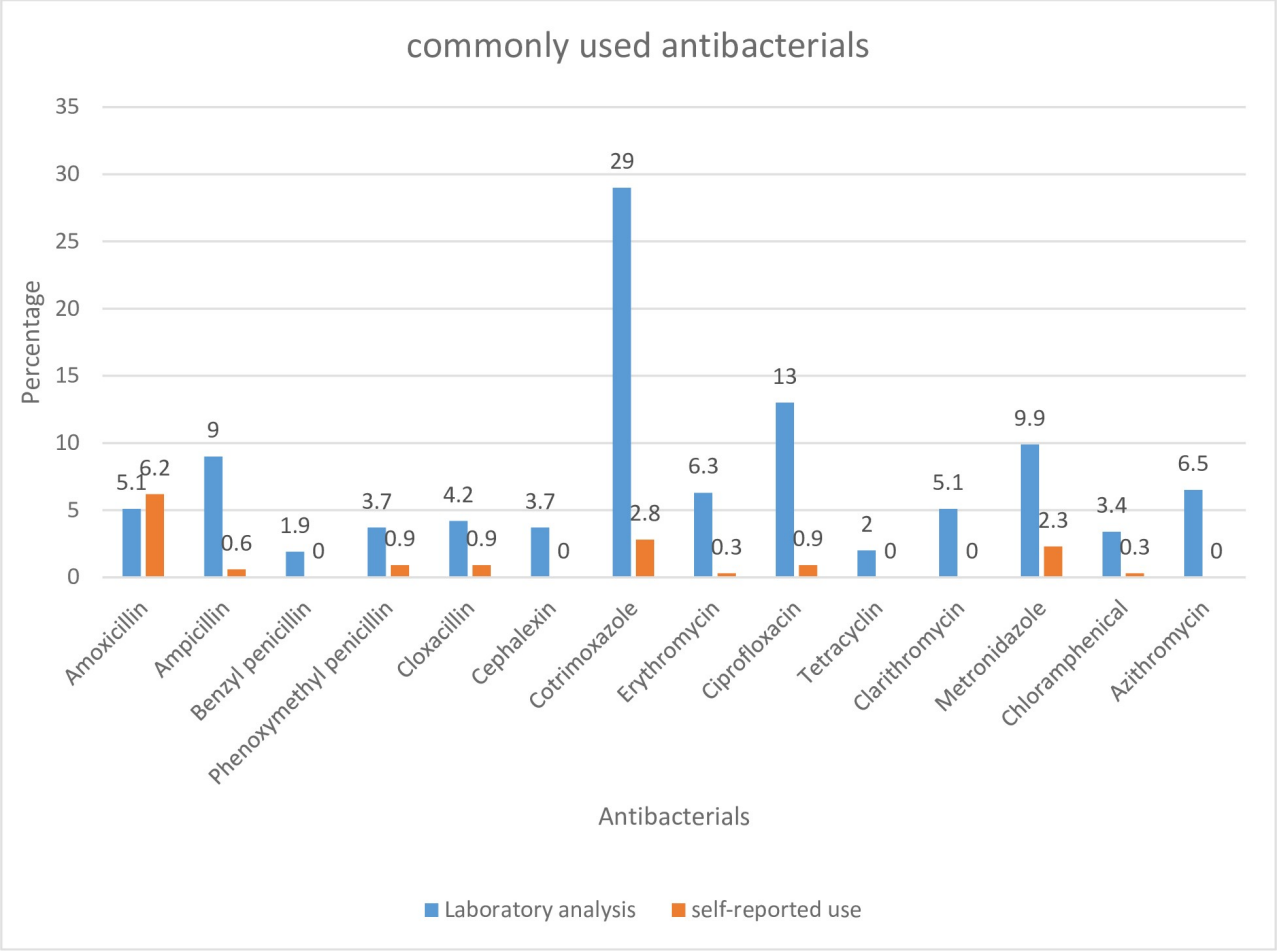
Commonly used antibacterials according to the laboratory analysis.

The most commonly used antibacterials as reported by the care givers were amoxicillin (6.2%, 22/355), cotrimoxazole (2.8%, 10/355), and metronidazole (2.3%, 8/355). The most common antibacterials detected from the laboratory analysis were cotrimoxazole (29%, 103/355), ciprofloxacin (13%, 46/355), and metronidazole (9.9%, 35/355) (Fig 1)

### Validity of caregivers’ reports of antibacterial intake in children under five years

The sensitivity, specificity, PPV, NPV, agreement and kappa coefficient of the caregivers’ reports of use of antibacterials for treatment of children prior to healthcare facilities visit were 17.3% (12.6–22.8), 90.7% (84.3–95.1), 76.5% (62.5–87.2), 38.5% (33.0–44.2), 43.9% (38.7–49.3%) and 0.06 (0.01–0.12) respectively. The sensitivity, specificity, PPV,NPV, agreement and kappa coefficient varied between the different antibacterials (see Table 3).

**Table 3:** Validity of caregivers’ reports of antibacterial intake in children under five years in rural communities of Gulu district, northern Uganda (August, 2019).

Parameters	Overall	Amoxicillin	Cotrimoxazole	Metronidazole	Ciprofloxacin
Sensitivity (95% CI)	17.3 (12.6–22.8)	5.6 (0.1–27.3)	5.8 (2.3–12.2)	2.9 (0.1–14.9)	4.3 (0.5–14.8)
Specificity (95% CI)	90.7 (84.3–95.1)	93.8 (90.6–96.1)	98.4 (96.0–99.6)	97.8 (95.5–99.1)	99.7 (98.2–100)
PPV (95% CI)	76.5 (62.5–87.2)	4.5 (0.1–22.8)	60.0 (26.3–87.8)	12.5 (0.3–52.7)	66.7 (9.4–99.2)
NPV (95% CI)	38.5 (33.0–44.2)	94.9 (92.0–97.0)	71.9 (66.8–76.6)	90.2 (86.6–93.1)	87.5 (83.6–90.8)
Prevalence (95% CI)	63.7 (58.4–68.7)	5.1 (3.0–7.9)	29.0 (24.3–34.0)	9.9 (7.0–13.4)	13.0 (9.6–16.9)
Agreement (95% CI)	43.9 (38.7–49.3)	89.3 (85.6–92.3)	71.5 (66.5–76.2)	88.5 (84.7–91.6)	87.3 (83.4–90.6)
κ (95% CI)	0.06 (0.01–0.12)	-0.01 (-0.11–0.09)	0.06 (-0.01–0.12)	0.01 (-0.08–0.1)	0.07 (-0.03–0.16)

%: percentage; CI: Confidence interval; κ: Kappa coefficient

## Discussion

In this study we demonstrated that the prevalence of antibacterial use prior to health facility visit was high and that caregivers under reported the use of antibacterials in the children under five years prior to coming to the health facility. Antibacterial use prior to healthcare facility visit is a common practice in many resource limited settings globally. Caregivers’ ability to report antibacterial use before coming to the health facility is crucial for appropriate prescription of antibacterial upon reaching health facilities [5]. Appropriate prescription of antibacterials is important because it reduces the emergence of antibacterial resistance, poor clinical outcomes, increased mortality and wastage of financial resources [19].

In the current study, almost two thirds (63.7%) of the samples (blood and/or urine) tested positive for antibacterials. This implies that the prevalence of antibacterial use prior to health facility visit is much higher than what was self-reported (14.4%). This finding is similar to those from other low and middle income countries (LMIC) [4, 5, 10], a study carried out in Ghana reported a prevalence of self-reported antibacterial use prior to health facility visit of 13%, however, analysis of urine samples reported a much higher prevalence of 64% [10]. In Uganda, self-medication with antibacterials is a common practice [1, 4, 20] which is reflected in the high prevalence of antibacterials found in the samples (blood and/or urine) in the current study [1]. Another reason for the high prevalence of antibacterial use in our study is the high prevalence of infectious diseases in these communities. In Uganda, 71% of children under five years attending healthcare facilities do so due to acute respiratory infections [21], however, in the community in this study, the most common diseases in children under five years seeking care at healthcare facilities include; malaria, diarrhea, pneumonia, acute childhood malnutrition and HIV/AIDS [15–17]. High prevalence of antibacterials found in the samples in the current study could also be due to exposure to antibacterials through consumption of water, vegetables and animal products [22, 23]. In the Hong Kong survey to determine the presence of veterinary antibiotics in food, drinking water, and the urine of preschool children, it was found that 13 veterinary antibiotics were detectable in the urine of 77.4% of primary school children with norfloxacin and penicillin having the highest detection rates. Enrofloxacin, penicillin, and erythromycin were the most detected veterinary antibiotics in raw and cooked food [21]. Studies in Uganda, report a high prevalence of veterinary use of antibacterials. The most commonly used antibacterials in veterinary medicine in Uganda include; procaine penicillin, trimethoprim/sulfadiazine, erythromycin sulphate, tylosin tartrate, oxytetracycline hydrochloride [24, 25]. This high prevalence of antibacterial use can lead to increased risk of resistance within the community [26]. A study was carried out in Uganda to determine the epidemiology and antibiotic susceptibility of Vibrio cholerae associated with the 2017 outbreak in Kasese district, and it reported that *V*. *cholerae* was highly resistant to the commonly used antibiotics [27].

Most caregivers reported to have given their children amoxicillin, cotrimoxazole and metronidazole. This is consistent with reports from a study in northern Uganda where metronidazole, amoxicillin, ciprofloxacin, doxycycline or cotrimoxazole were reported as the most commonly used antibacterials by patients prior to hospital visit [4]. Metronidazole is commonly used for bacterial gastroenteritis, amoxicillin is used for bacterial chest infections, and cotrimoxazole is used to treat pneumonia, bronchitis, infections of the urinary tract, ears intestines and as prophylaxis against opportunistic infections in HIV [28, 29]. In our study the most commonly detected antibacterials in the laboratory analysis results were cotrimoxazole, ciprofloxacin and metronidazole, similar to findings from a study carried out in Ghana which reported ciprofloxacin, trimethoprim or metronidazole as the most common antibacterials detected in urine samples [30]. Ciprofloxacin is commonly used to treat pneumonia, typhoid fever, infectious diarrhea, skin and bone infections [28, 29]. Amoxicillin was the most commonly reported antibacterial used and yet it was not among the most commonly detected antibacterials from the laboratory analysis. This could be explained by the pharmacokinetics of amoxicillin, which has a very short half-life of about 1 hour and will usually be out of the system within 5 hours. Thus, meaning that for it to be detected in the blood or urine samples, it should have been taken within a few hours before healthcare facility visit [28, 29]. We also observed that the number of children who had cotrimoxazole in their biological samples was higher than those who reported the use. It is possible that some of these children may have tested positive for cotrimoxazole since they could have been receiving it as prophylaxis against opportunistic infections in HIV [31, 32]. The prevalence of HIV/AIDS in northern Uganda as of 2019 when data for this study was collected, was 7.2% in adults and 0.5% in children under five years [33]. Since we were interested in antibacterial use for current illness, for which the children were brought to the healthcare facility, caregivers might not have found it not necessary to report the use of cotrimoxazole as prophylaxis against opportunistic infections in HIV.

The positive predicative value we found for reported use of antibacterials is not high enough to allow caregivers reports to guide treatment. The high specificity values indicate under reporting but the negative predictive value indicate that many children were given drugs that were not reported by caregivers. This study was carried out in rural communities of Gulu district in Uganda where the adult literacy levels are low [1, 20], and the inconsistencies in caregivers’ response to interview questions and laboratory findings, could have been because of caregivers inability to identify medicines taken as antibacterials. Another reason for the inconsistencies in self-reported antibacterial use and laboratory findings could have been due to social desirability bias [34]. The caregivers could have been aware that self-medication is not a good practice, and therefore feared to tell the interviewers the truth. Another reason for the inconsistencies could have been due to consumption of these medicines from diffuse sources such as milk, water or food, studies in Uganda have reported veterinary use of antibacterials [24, 25]. Another worrying explanation for the inconsistencies could be the quality of antibacterial medicines, some of these antibacterials may not contain the actual quantity of the active medicine the manufacturers claim they contain. Although we did not set out to study the quality of antibacterials in this study, high prevalence of substandard antibacterial medicines has been previously reported in developing countries [35]. Furthermore, inaccuracies in self-reports may lead to duplication of therapy, incorrect management of the ill child, failure to appreciate non-compliance leading to exacerbation of chronic medical conditions, or inaccurate research conclusions [36].

We observed a strong association between high self-reported prior antibacterial use and the source of antibacterials being from public health facilities. This could be attributed to the low financial status of the people in these communities [1] forcing them to seek free healthcare from public healthcare facilities. The district-based healthcare system in Uganda consists of level I, II, III, IV and district hospitals [11]. This therefore means that by the time these children were brought to health care level III and IV, they could have already sought care from the lower levels and were referred to these higher levels for further management.

There is need for further research to understand the reasons for caregivers’ poor reports on their children’s prior intake of antibacterials before coming to the health facility. Improved validity could be promoted by encouraging health care workers to carefully explain to the caregivers the medicines they administer to these children when they fall sick. Proper documentation of the medicines given to these children when they are sick could also improve the validity of self-reported medicine use. There is need for the healthcare workers to educate the caregivers about the dangers of using antibacterials without consulting a healthcare worker, and also further research is required to better understand why caregivers initiate antibacterial use at home without consulting a healthcare service provider. This all will allow policy makers to be better informed when planning interventions to reduce the large amount of incorrect antibacterial use in the community.

The results of our study should be considered in light of some limitations. This study could have been affected by recall bias, where antibacterials given may have been forgotten. The study could have also been affected by social desirability bias since the study was carried out in a hospital setting and probably caregivers feared telling the truth because they thought it could affect patient care. Under reporting could have been affected by how the questions were understood by the caregivers. Failure to detect some of the antibacterials in the samples could have been due to the pharmacokinetics of the antibacterials. Factors such as education level of the caregivers could have contributed to the under reporting of antibacterial use prior to healthcare facility visit, however, we didn’t collect this information. This is because adult literacy levels in this community are low [1, 20] and to our knowledge previous studies have not reported associations between self-report and education level [10, 37]. However, there is need for further research to determine if there’s an association between caregivers’ education level and reporting of prior antibacterial use in this setting. The discrepancy between the reported use and the detected antibacterials in blood/urine samples could have also been because the antibacterial could have been given for the management of another condition, such as cotrimoxazole for prophylaxis against HIV related opportunistic infections, but we did not collect this information. Our tool was designed to capture only antibacterial use for the illness for which the children were brought to the healthcare facility. We were unable to report the levels of antibacterials in relation to how far back the antibacterials were taken, this is because we used a qualitative LC-MS/MS method which was developed to report the presence or absence of antibacterials and not to quantify them.

## Conclusion

A high proportion of children under five years take antibacterials prior to visiting a healthcare facility in northern Uganda. However, there is low validity of caregivers’ reports on prior intake of antibacterials by these children. There is need for further research to understand the factors associated with under reporting of prior antibacterial medicine use by caregivers of children under five years. In addition, we suggest that health care workers should endeavor to explain the role and names of medicines during dispensing, as well as the importance of reporting correctly on prior medication intake. There is also need to educate the caregivers about the dangers of using antibacterials without consulting a healthcare worker, and also further research is required to better understand why caregivers initiate antibacterial use at home without consulting a healthcare service provider. This all will allow policy makers to be better informed when planning interventions to reduce the large amount of incorrect antibacterial use in the community.

## Supporting information

S1 Table Summary of key pharmacokinetic properties of some of the antibacterials that are commonly used among children under five years in rural communities of Gulu district, northern Uganda (August, 2019). (DOCX)

S1 Appendix Ethical approval letters. (DOCX)

S2 Appendix Consent form. (DOCX)

S3 Appendix LC-MS/MS method. (DOCX)

S4 Appendix Questionnaire. (DOCX)
