# Supplementary material for: Validity of caregivers’ reports on prior use of antibacterials in children under five years presenting to health facilities in Gulu, northern Uganda
Source: PLoS One. 2021 Sep 16;16(9):e0257328. doi: 10.1371/journal.pone.0257328 (PMC8445424; doi:10.1371/journal.pone.0257328)
Supplement: S1 Table — (DOCX) [file pone.0257328.s001.docx]

**S1 Table. Summary of key pharmacokinetic properties of some of the antibacterials that are commonly used among children under five years in rural communities of Gulu district, northern Uganda (August, 2019).**

| **Antibacterial agent** | **Time to peak plasma concentrations**  **(hours)** | **Volume of distribution**  **(L/kg)** | **Urinary excretion (%)** | **Clearance (mL/min/kg)** | **Terminal Half-life (hours)** | **Reference** |
| --- | --- | --- | --- | --- | --- | --- |
| Amoxicillin | 1-2 | 0.21 ± 0.03  ↔ RD, Aged | 86 ± 8 | 2.6 ± 0.4  ↑ Pregnancy, ↓ RD, Aged  ↔ child | 1.7 ± 0.3  ↑ RD, Aged  ↔ child | [1-3] |
| Ampicillin | 1-2 | 0.22-0.39 | 75-85 | 2.8 | 1-1.8  ↑ RD | [4-9] |
| Penicillin G | PO: 0.5-1  IM, short acting: 0.5  IM, Procaine: 1-3  IM, Benzathine: 12-24 | 0.33 | 79 | 5-9  ↓ RD | 0.5 ± 0.1  ↑ RD | [10-13] |
| Penicillin V | 0.5-1 | 0.41 | 25  ↓ RD, neonate, child | 6.8 | 0.5-0.6  ↑ RD | [7, 9, 14] |
| Cloxacillin | 0.58-1 | 0.3 | 37 (500mg oral dose)  62 (750mg IV dose)  78 excreted unchanged in urine | 1.6 ± 0.3  ↓ RD | 0.5  ↑ RD | [15-19] |
| Erythromycin | 2.1-3.9  (250-mg oral enteric-coated free base in a capsule);  2-3  (250-mg film-coated tablet or capsule of erythromycin stearate) | 0.78 ± 0.44  ↑ RD | 12 ± 7 | 9.1 ± 4.1 | 1.6 ± 0.7 | [20] |
| Ciprofloxacin | 0.6 ± 0.2 | 2.2 ± 0.4  ↓ Aged | 50 ± 5 | 7.6 ± 0.8  ↓ RD, Aged | 3.3 ± 0.4  ↑ RD | [21, 22] |
| Chloramphenicol | 1-2 | 0.6-1 (oral) | 90 with 15% excreted unchanged | 2.4 | 1.5-4.6  ↑ RD, LD, Child | [7, 23-26] |
| Sulfamethoxazole | 4 | 0.26 ± 0.04  ↑ RD | 14 ± 2 | 0.31 ± 0.07 | 10.1 ± 2.6  ↑ RD | [27, 28] |
| Trimethoprim | 2 | 1.6 ± 0.2  ↑ Neonate, Child | 63 ± 10 | 1.9 ± 0.3  ↓ RD  ↑ Child | 10 ± 2  ↑ RD  ↓ Child | [27, 28] |
| Metronidazole | Orally: 2.8  Vaginal cream: 11 ± 2 | 0.74 ± 0.10 | 10 ± 2 | 1.3 ± 0.3  ↓ LD, Neonate | 8.5 ± 2.9  ↑ Neonate, LD | [29] |
| Azithromycin | 2-3 | 31 | 12 | 9 | 40  ↔ hepatic cirrhosis | [30] |
| Tetracycline | Oral: 4 | 1.5 ± 0.1 | 58 ± 8 | 1.67 ± 0.24 | 10.6 ± 1.5 | [31, 32] |
| Clarithromycin | 2.8  (clarithromycin);  0.7  (14-hydroxyclarithromycin) | 2.6 ± 0.5  ↑ LD | 36 ± 7 | 7.3 ± 1.9  ↓ Aged, RD | 3.3 ± 0.5 | [33, 34] |
| Cephalexin | 1.4 ± 0.8 | 0.26 ± 0.03 | 91 ± 18 | 4.3 ± 1.1  ↓ RD | 0.90 ± 0.18  ↑ RD | [35] |
| **L/kg: Liters per kilogram; mL/min/kg: milliliters/ minute/kilogram; %: percentage; RD: Renal Disease; LD: Liver Disease; PO: Per os (oral); IM: Intramuscular; IV: Intravenous; ↓: Reduced; ↑: Increased; ↔: Same as or equivalent** | | | | | | |

**References**

1. Höffler D. [The pharmacokinetics of amoxicillin]. Advances in clinical pharmacology. 1974;7:28-30.
2. Sjövall J, Alván G, Huitfeldt B. Intra- and inter-individual variation in pharmacokinetics of intravenously infused amoxycillin and ampicillin to elderly volunteers. Br J Clin Pharmacol. 1986;21(2):171-81.
3. Andrew MA, Easterling TR, Carr DB, Shen D, Buchanan ML, Rutherford T, et al. Amoxicillin pharmacokinetics in pregnant women: modeling and simulations of dosage strategies. Clinical pharmacology and therapeutics. 2007;81(4):547-56.
4. Nawaz M, Tabassum R, Iqbal T, Perveen Z. Disposition kinetics, renal clearance and excretion of ampicillin after oral administration in goats. Zentralblatt fur Veterinarmedizin Reihe A. 1990;37(4):247-52.
5. Ehrnebo M, Nilsson SO, Boréus LO. Pharmacokinetics of ampicillin and its prodrugs bacampicillin and pivampicillin in man. J Pharmacokinet Biopharm. 1979;7(5):429-51.
6. Dajani A. Use of ampicillin/sulbactam and sultamicillin in pediatric infections: a re-evaluation. The Journal of international medical research. 2001;29(4):257-69.
7. Knox C, Law V, Jewison T, Liu P, Ly S, Frolkis A, et al. DrugBank 3.0: a comprehensive resource for ‘omics’ research on drugs. Nucleic acids research. 2010;39(suppl_1):D1035-D41.
8. Blum RA, Kohli RK, Harrison NJ, Schentag JJ. Pharmacokinetics of ampicillin (2.0 grams) and sulbactam (1.0 gram) coadministered to subjects with normal and abnormal renal function and with end-stage renal disease on hemodialysis. Antimicrob Agents Chemother. 1989;33(9):1470-6.
9. Bergan T. Pharmacokinetics of beta-lactam antibiotics. Scandinavian journal of infectious diseases Supplementum. 1984;42:83-98.
10. Dittert LW, Griffen WO, Jr., LaPiana JC, Shainfeld FJ, Doluisio JT. Pharmacokinetic interpretation of penicillin levels in serum and urine after intravenous administration. Antimicrob Agents Chemother (Bethesda). 1969;9:42-8.
11. Ebert SC, Leggett J, Vogelman B, Craig WA. Evidence for a slow elimination phase for penicillin G. The Journal of infectious diseases. 1988;158(1):200-2.
12. McDermott W, Bunn PA, Benoit M, Dubois R, Reynolds ME. THE ABSORPTION, EXCRETION, AND DESTRUCTION OF ORALLY ADMINISTERED PENICILLIN. The Journal of clinical investigation. 1946;25(2):190-210.
13. Kucers A, Bennett NM, Company JBL. The use of antibiotics : a comprehensive review with clinical emphasis. Philadelphia: J. B. Lippincott Company; 1987.
14. Overbosch D, Mattie H, van Furth R. Comparative pharmacodynamics and clinical pharmacokinetics of phenoxymethylpenicillin and pheneticillin. Br J Clin Pharmacol. 1985;19(5):657-68.
15. Bodey GP, Vallejos C, Stewart D. Flucloxacillin: a new semisynthetic isoxazolyl penicillin. Clinical pharmacology and therapeutics. 1972;13(4):512-5.
16. Rosenblatt JE, Kind AC, Brodie JL, Kirby WM. Mechanisms responsible for the blood level differences of isoxazolyl penicillins: oxacillin, cloxacillin, and dicloxacillin. Archives of internal medicine. 1968;121(4):345-8.
17. Nauta EH, Mattie H. Pharmacokinetics of flucloxacillin and cloxacillin in healthy subjects and patients on chronic intermittent haemodialysis. Br J Clin Pharmacol. 1975;2(2):111-21.
18. Eadie MJ. Neurological clinical pharmacology / Mervyn J. Eadie, John H. Tyrer. Tyrer JH, editor. New York ; Sydney: Adis Press; 1980.
19. Nauta EH, Mattie H. Dicloxacillin and cloxacillin: pharmacokinetics in healthy and hemodialysis subjects. Clinical pharmacology and therapeutics. 1976;20(1):98-108.
20. Periti P, Mazzei T, Mini E, Novelli A. Clinical pharmacokinetic properties of the macrolide antibiotics. Effects of age and various pathophysiological states (Part I). Clin Pharmacokinet. 1989;16(4):193-214.
21. Begg EJ, Robson RA, Saunders DA, Graham GG, Buttimore RC, Neill AM, et al. The pharmacokinetics of oral fleroxacin and ciprofloxacin in plasma and sputum during acute and chronic dosing. Br J Clin Pharmacol. 2000;49(1):32-8.
22. Sörgel F, Jaehde U, Naber K, Stephan U. Pharmacokinetic disposition of quinolones in human body fluids and tissues. Clin Pharmacokinet. 1989;16 Suppl 1:5-24.
23. Ambrose PJ. Clinical pharmacokinetics of chloramphenicol and chloramphenicol succinate. Clin Pharmacokinet. 1984;9(3):222-38.
24. Narang AP, Datta DV, Nath N, Mathur VS. Pharmacokinetic study of chloramphenicol in patients with liver disease. Eur J Clin Pharmacol. 1981;20(6):479-83.
25. Kurz H, Mauser-Ganshorn A, Stickel HH. Differences in the binding of drugs to plasma proteins from newborn and adult man. I. Eur J Clin Pharmacol. 1977;11(6):463-7.
26. Shepherd G. Chloramphenicol. In: Wexler P, editor. Encyclopedia of Toxicology (Second Edition). New York: Elsevier; 2005. p. 538-9.
27. Hutabarat RM, Unadkat JD, Sahajwalla C, McNamara S, Ramsey B, Smith AL. Disposition of drugs in cystic fibrosis. I. Sulfamethoxazole and trimethoprim. Clinical pharmacology and therapeutics. 1991;49(4):402-9.
28. Welling PG, Craig WA, Amidon GL, Kunin CM. Pharmacokinetics of trimethoprim and sulfamethoxazole in normal subjects and in patients with renal failure. The Journal of infectious diseases. 1973;128:Suppl:556-66 p.
29. Lau AH, Lam NP, Piscitelli SC, Wilkes L, Danziger LH. Clinical pharmacokinetics of metronidazole and other nitroimidazole anti-infectives. Clin Pharmacokinet. 1992;23(5):328-64.
30. Lalak NJ, Morris DL. Azithromycin clinical pharmacokinetics. Clin Pharmacokinet. 1993;25(5):370-4.
31. Raghuram TC, Krishnaswamy K. Pharmacokinetics of tetracycline in nutritional edema. Chemotherapy. 1982;28(6):428-33.
32. Garty M, Hurwitz A. Effect of cimetidine and antacids on gastrointestinal absorption of tetracycline. Clinical pharmacology and therapeutics. 1980;28(2):203-7.
33. Chu SY, Deaton R, Cavanaugh J. Absolute bioavailability of clarithromycin after oral administration in humans. Antimicrob Agents Chemother. 1992;36(5):1147-50.
34. Fraschini F, Scaglione F, Demartini G. Clarithromycin clinical pharmacokinetics. Clin Pharmacokinet. 1993;25(3):189-204.
35. Spyker DA, Thomas BL, Sande MA, Bolton WK. Pharmacokinetics of cefaclor and cephalexin: dosage nomograms for impaired renal function. Antimicrob Agents Chemother. 1978;14(2):172-7.
