# Supplementary material for: Validity of caregivers’ reports on prior use of antibacterials in children under five years presenting to health facilities in Gulu, northern Uganda
Source: PLoS One. 2021 Sep 16;16(9):e0257328. doi: 10.1371/journal.pone.0257328 (PMC8445424; doi:10.1371/journal.pone.0257328)
Supplement: S1 Appendix — (DOCX) [file pone.0257328.s002.docx]

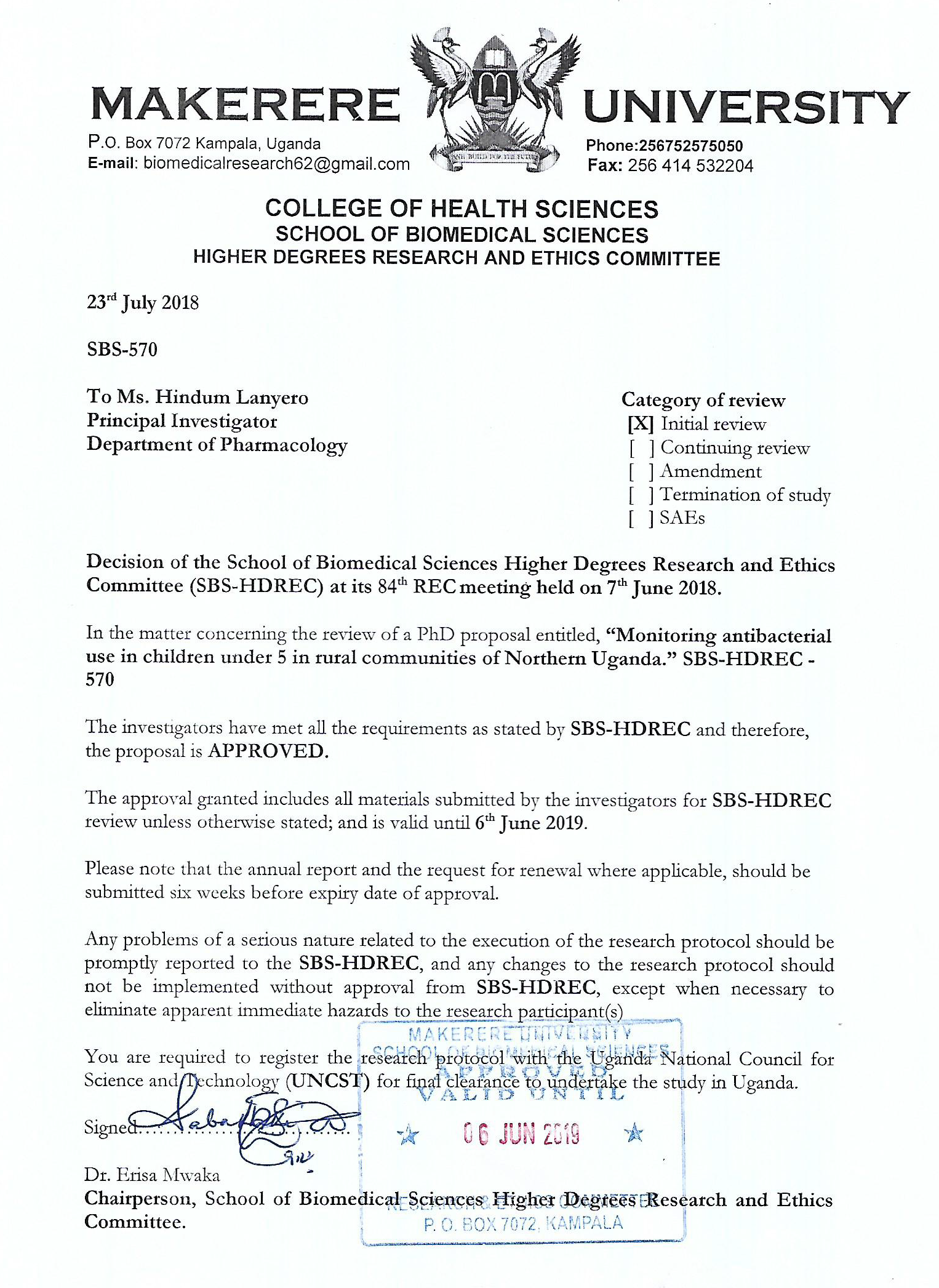
**Appendix 1: Ethical approval letters**

|  |
| --- |
|  |
| \|  \| \| --- \| \| Dear Hindum Lanyero,  I am pleased to inform you that on **06/11/2018**, the Uganda National Council for Science and Technology (UNCST) approved your study titled, **Monitoring antibacterial use in children under five in rural Communities of northern Uganda.**. The Approval is valid for the period of **06/11/2018** to **06/11/2021**.  Your study reference number is **HS235ES**. Please, cite this number in all your future correspondences with UNCST in respect of the above study.  Please, note that as Principal Investigator, you are responsible for:   1. Keeping all co-investigators informed about the status of the study. 2. Submitting any changes, amendments, and addenda to the study protocol or the consent form, where applicable, to the designated local Research Ethics Committee (REC) or Lead Agency, where applicable, for re-review and approval prior to the activation of the changes. 3. Notifying UNCST about the REC or lead agency approved changes, where applicable, within five working days. 4. For clinical trials, reporting all serious adverse events promptly to the designated local REC for review with copies to the National Drug Authority. 5. Promptly reporting any unanticipated problems involving risks to study subjects/participants to the UNCST. 6. Providing any new information which could change the risk/benefit ratio of the study to the UNCST for review. 7. Submitting annual progress reports electronically to UNCST. Failure to do so may result in termination of the research project.   Please, note that this approval includes all study related tools submitted as part of the application.  Yours sincerely, Hellen Opolot For: Executive Secretary **UGANDA NATIONAL COUNCIL FOR SCIENCE AND TECHNOLOGY** \| |
|  |
