# Supplementary material for: Validity of caregivers’ reports on prior use of antibacterials in children under five years presenting to health facilities in Gulu, northern Uganda
Source: PLoS One. 2021 Sep 16;16(9):e0257328. doi: 10.1371/journal.pone.0257328 (PMC8445424; doi:10.1371/journal.pone.0257328)
Supplement: S2 Appendix — (DOCX) [file pone.0257328.s003.docx]

**Appendix 2: Consent form**

**INFORMED CONSENT FORM FOR SUB-STUDY 3(Consent will be got from care givers of children under five).**

**Title of the proposed study:** MONITORING ANTIBACTERIAL USE IN CHILDREN UNDER FIVE IN RURAL COMMUNITIES OF NORTHERN UGANDA.

**Investigators :**

1. Dr. Hindum Lanyero (Principle investigator) Makerere University College of Health Science.
2. Dr. Sarah Nanzigu (Supervisor) Makerere University College of Health Science.
3. Dr. Moses Ocan (Supervisor) Makerere University College of Health Science.
4. Dr. Jaran Eriksen (Supervisor) Karolinska Institutet.

**Background and rationale for the study:**

High prevalence of antibacterial medicine use has been reported in children especially those under-five, these children are often treated inappropriately. This situation is likely to affect treatment outcomes as well as increase resistance development, drug toxicity and other adverse effects. The high prevalence of antibacterial use coupled with inappropriate medicine use poses threats to measures which have been put in place to make sure that medicines are used appropriately. These measures require regular and objective monitoring but this is often not done especially in children under five, even the methods used for this kind of studies have not been well researched in low resource settings like Uganda. Self-reports that are often utilized in such studies have been found to have low validity thereby necessitating the use of more objective monitoring tools.

**Purpose:**

The purpose of this study is to monitor antibacterial use in children who develop symptoms of acute upper respiratory tract infections and/or diarrhea in rural communities of northern Uganda and develop a method to validate the reported use.

**Procedures:**

The study will involve you answering questions on the kind of treatment that your child received before coming to the health facility. Blood and urine samples will be collected from your child and taken to the laboratory for analysis to this see which medicines they contain. The blood sample will be collected by a finger prick and then urine will be collected in urine bags for babies or plastic cups for big children.

**Who will participate in the study?**

Care givers of children under five who will give information about antibacterial medicines taken before coming to health facility and children under five from whom blood and urine samples will be collected.

**Risks/Discomfort:**

The study is not risky since only a small blood sample will be collected by finger prick.

**Benefits:**

Your participation will be of great importance in improving health service delivery to individuals in relation to drug use.

The information we get from this study will help contribute to efforts towards mitigation of antibacterial misuse through evidence- based interventions that will not only contribute to reduction in morbidity and mortality but also lead to reduction in wastage of financial resources.

**Confidentiality:**

Your involvement is appreciated and confidentiality will be highly observed, your identification will not be required to appear anywhere in the study and only the principle investigator will have access to your telephone number.

**Alternatives:**

Your participation is voluntary and you have a right to refuse answering any question that you feel uncomfortable with, you are also free to withdraw from the study at any time without fear of any consequences.

**Costs:**

You will not incur any costs in terms of money by participating in this study

**Compensation for participation:**

You will be given 10,000 Ugandan shillings for your lunch (snack and soda).

**Reimbursement:**

You will not be given any transport refund given that you had come to the health facility for treatment and volunteered to participate in the study that was ongoing in the facility.

**Questions:**

In case you have any questions that you want to ask or any additional information you want to give regarding this study after today you can reach the principle investigator, Dr. Hindum Lanyero on **0775866653.**

**Questions about participants rights:**

In case you have any questions that you want to ask about your rights, you can call the chair, School of Biomedical Sciences Higher Degrees Research and Ethics Committee on **0414533541.**

**Consent:**

By signing below, you indicate that you have understood the information presented to you and that you voluntarily give your consent to participate in the study.

Signature of respondent………………………. Tel no:.......................... Date…………………..

Signature of researcher……………………….. Date…………………….
