# Supplementary material for: Validity of caregivers’ reports on prior use of antibacterials in children under five years presenting to health facilities in Gulu, northern Uganda
Source: PLoS One. 2021 Sep 16;16(9):e0257328. doi: 10.1371/journal.pone.0257328 (PMC8445424; doi:10.1371/journal.pone.0257328)
Supplement: S3 Appendix — (DOCX) [file pone.0257328.s004.docx]

**Appendix 3: LC-MS/MS method**

**Chromatographic system**

Instrumentation separation of analytes for this method was performed using an Agilent 1290 infinity II LC with a 20 µL injection port and multiwash capability. An Agilent 6420 Triple Quadrupole LC/MS was used as the detector. Electrospray ionization was performed in simultaneous positive and negative mode. All data acquisition and processing were performed using Agilent MassHunter software (version B 07.00). Table 1 below shows the LC-MS/MS instrument conditions and table 2 shows the different MRM transitions used for the different antibiotics.

**Table 1:LC-MS/MS instrument conditions**

| **Optimized conditions** | | |
| --- | --- | --- |
| **Parameter** | **Value** | |
| Instrument | Agilent 1290 Infinity II with 20 µL flex loop and multiwash | |
| Column | Agilent ZORBAX C-18 Eclipse Plus 2.1 × 150 mm, 1.8 µm (p/n 959759-902) | |
| Column temperature | 45^0^C | |
| Injection volume | 10 µL | |
| Mobile phase | Mobile phase  A) Water + 0.1 % formic acid +0.1%ammonium formate  B) 80% acetonitrile+20% methanol+ 0.1% formic acid | |
| Run time | 25 minutes | |
| Post run time | 2 minutes | |
| Flow rate | 0.25 mL/min | |
| Gradient | Time (minutes) B (%) A(%)  0 2.5 97.5  1 2.5 97.5  3 5.0 95.0  5.0 30.0 70.0  8.0 50.0 50.0  12.0 70.0 30.0  17.0 95.0 5.0  20.0 100.0 0.0  22.0 100.0 0.0  22.2 2.5 97.5  25.0 2.5 97.5 | |
| Mass spectrometer | | Agilent 6420 Triple Quadrupole LC/MS |
| Gas temperature | | 330^0^C |
| Gas flow rate | | 11 L/min |
| Nebulizer pressure | | 30 psi |
| Capillary voltage | | 4000 V (3000V) |
| DELTA EMV | | 200 V |

**Table 2: MRM transitions used for the detection of the different antibiotics**

| **Analyte** | **Major** | **CE (V)** | **Minor** | **CE (V)** | **Polarity** |
| --- | --- | --- | --- | --- | --- |
| Azithromycin | 749.5/591.4 | 20 | 749.5/158.1 | 40 | Positive |
| Clarithromycin | 748.5/590.4 | 20 | 748.5/158.1 | 20 | Positive |
| Erythromycin | 734.5/576.4 | 20 | 734.5/158.1 | 40 | Positive |
| Tetracycline | 445.2/427.1 | 10 | 445.2/410.1 | 20 | Positive |
| Cloxacillin | 436/277 | 15 | 436/160 | 15 | Positive |
| Amoxicillin | 366.1/349.1 | 10 | 366.1/114 | 20 | Positive |
| Penicillin V | 351/192 | 5 | 351/160 | 10 | Positive |
| Cephalexin | 348.24/158.1 | 15 | 348.24/106.2 | 25 | Positive |
| Penicillin G | 335.1/176.1 | 10 | 335.1/160 | 10 | Positive |
| Ciprofloxacin | 332.1/314.1 | 20 | 332.1/231.1 | 40 | Positive |
| Trimethoprim | 291.1/230.1 | 20 | 291.1/123.1 | 40 | Positive |
| Sulfamethoxazole | 254.1/156 | 10 | 254.1/92 | 20 | Positive |
| Metronidazole | 172.1/128 | 10 | 172.1/82.1 | 20 | Positive |
| Ampicillin | 348.1/207.1 | 10 | 348.1/74 | 40 | Negative |
| Chloramphenicol | 321/257 | 10 | 321/152 | 20 | Negative |

**Limit of detection**

The limit of detection for the different antibacterials were: amoxicillin (1.34 ng/mL), ampicillin (0.001 ng/mL), penicillin G (0.005 ng/mL), penicillin V (0.03 ng/mL), cloxacillin (0.2 ng/mL), cephalexin (0.22 ng/mL), sulfamethoxazole (0.95 ng/mL), trimethoprim (0.52 ng/mL), erythromycin (1.1 ng/mL), ciprofloxacin (0.1 ng/mL), tetracycline (0.14 ng/mL), clarithromycin (1.4 ng/mL), metronidazole (0.0004 ng/mL), chloramphenicol (0.0001ng/mL) and azithromycin (0.22 ng/mL).
