# Supplementary material for: Validity of caregivers’ reports on prior use of antibacterials in children under five years presenting to health facilities in Gulu, northern Uganda
Source: PLoS One. 2021 Sep 16;16(9):e0257328. doi: 10.1371/journal.pone.0257328 (PMC8445424; doi:10.1371/journal.pone.0257328)
Supplement: S4 Appendix — (DOCX) [file pone.0257328.s005.docx]

**Appendix 4: Questionnaire**

# Questionnaire for collecting antibacterial drug use for sub-study III

**Questionnaire for collecting data on drugs taken before coming to the health care facility**

**Sub-study 3**

Health center interview guide

Date………………………………………………………….

Name of Health facility…………………………………………….

Tel contact of respondent/home…………………………………

Sub-county code…………………………………………..

Sex……………………………………..Age……………………………………

1 Did you give the child any medicines to take for this illness before coming to the health care ? (a) Yes  (b) No 

2. (a) If yes, which medicine did you give the child?

| **No** | **Medicine**  Write name of medicine  *If name is not known write the most detailed category given by respondent e.g antibacterial, for fever, or description* | **Formulation**  1 tablet  2 capsule  3vials/injection  4cream/ ointment  5Eye/ear drop  6 Syrup  7 other(specify) | **Recommended/prescribed by**  1. care taker  2. other household member  3. Friend/neighbor  4. Doctor/nurse  5. Drug seller/Pharmacist  6. Traditional healer | **Obtained from**  1. Home cabinet  2. Public health facility  3. Private clinics  4. Drug shop  5. Retail shops  6. Traditional healer |
| --- | --- | --- | --- | --- |
| 1 |  |  |  |  |
| 2 |  |  |  |  |
| 3 |  |  |  |  |
| 4 |  |  |  |  |
| 5 |  |  |  |  |
| 6 |  |  |  |  |
| 7 |  |  |  |  |
| 8 |  |  |  |  |
| 9 |  |  |  |  |
| 10 |  |  |  |  |

(b) How did you take the medicines that you used to treat the child before coming to the facility? Fill the table below.

| Drug use | Monday | Tuesday | Wednesday | Thursday | Friday | Saturday | Sunday |
| --- | --- | --- | --- | --- | --- | --- | --- |
| What I used |  |  |  |  |  |  |  |
| Amount I used |  |  |  |  |  |  |  |
| When |  |  |  |  |  |  |  |
| Where |  |  |  |  |  |  |  |
| with |  |  |  |  |  |  |  |
| why |  |  |  |  |  |  |  |

(c) When was last dose given? …..............................
